# Efficacy of Intravenous Iron Infusion in Gestational Iron Deficiency Anemia in Dubai, United Arab Emirates, During 2018–2019: A Retrospective Study

**DOI:** 10.7759/cureus.60185

**Published:** 2024-05-13

**Authors:** Hamda A Alfalasi, Shabana Kausar

**Affiliations:** 1 Medicine, Mohammed Bin Rashid University, Dubai, ARE; 2 Obstetrics and Gynecology, Mediclinic City Hospital, Dubai, ARE

**Keywords:** oral iron pregnancy, intravenous iron replacement, iron supplementation, gestational iron deficiency anemia, iron deficiency anemia (ida)

## Abstract

Objective: The objective is to investigate the effectiveness of intravenous (IV) iron infusion in increasing hemoglobin levels in gestational iron deficiency anemia (GIDA) patients in a tertiary-care hospital in Dubai emirate, United Arab Emirates (UAE).

Methods: This is a retrospective cohort study of GIDA patients who were exposed to IV iron infusion supplementation. Study data of 40 cases aged 25-45 in a tertiary-care hospital in the UAE between 2018 and 2019 were analyzed. Variables accounted for were maternal age, age of gestation when IV iron was administered, and IV iron dose.

Results: The average hemoglobin level before the intervention was 9 g/dL, and the average change after the intervention was 10.4 g/dL with a mean of 1.4 g/dL difference between before and after the intervention.

Conclusion: Supplementation of IV iron infusion in GIDA patients was seen to have increased the hemoglobin level after the intervention; however, the increase did not meet the recommended range of 12-16 g/dL.

## Introduction

Background

Anemia is a condition in which the number of red blood cells (RBCs) or the hemoglobin concentration within them is lower than normal [1]. The average Hb level for males is 14 to 18 g/dL; that for females is 12 to 16 g/dL. Many pregnant women become anemic due to iron deficiency; about 30% of pregnant women are affected in developed countries, and over 50% in developing countries [2]. By cause of pregnancy, a female’s body requires more blood to support the fetus’s growth, which requires a good source of nutrients. When the body does not meet that, the patient becomes anemic.

Patients with gestational iron deficiency anemia (GIDA) may present with symptoms of fatigue, dizziness, and impaired immune response predisposing to infections [3]. Before pregnancy, women tend to have low or empty iron stores. A large French study, which included a total of 6,648 women, showed depleted iron stores (serum ferritin <15 μg/L) in one out of five women (22.7%) of childbearing age [4]. To prevent such depletion, they must be screened throughout their pregnancy for any changes in hemoglobin levels [5]. However, when they are prescribed to take oral iron supplements to prevent it, they are non-compliant. In the Yaounde gyneco-obstetric and pediatric hospital, the adherence of their patients to iron supplementation was assessed, where it was concluded that in 304 recruited women, 16.4% were highly compliant, and 27.6% were moderately compliant. In contrast, 56% had low compliance with iron supplementation during pregnancy [6]. A systematic review and a meta-analysis study were performed at Ethiopia’s national level, where the results stated that “more than four of nine pregnant women have adhered to the iron and folic acid supplementation” [7]. Thus, by the time they are in their 24th-30th gestational week, they become at risk of premature labor [8], preeclampsia [9], placental abruption [10], low birth weight, birth asphyxia, and neonatal anemia [11]. Anemia is also the ‘leading cause of infancy iron deficiency anemia’ [12].

Anemia has also been associated with increased morbidity and mortality rates in postpartum. When a patient is diagnosed with GIDA, they would have a reduced ability to compensate for the bleeding loss that occurs during labor, whether it is a vaginal or cesarean section form of delivery. It is an increased risk in the latter [13,14]. The mainstay treatment is RBC transfusion [15]. However, this only corrects the blood loss rather than the underlying cause of iron deficiency anemia [16,17].

A retrospective study assessed the effectiveness, tolerability, and safety of ferric carboxymaltose administered intravenously in managing iron deficiency anemia in pregnant women in Abu Dhabi, UAE. It was concluded that 41.4% of the women achieved an increase of ≥2 g/dL in blood hemoglobin [18]. However, in this study, the effectiveness of intravenous (IV) iron in raising hemoglobin levels in pregnant patients with severe anemia or noncompliance with oral iron supplementation will be investigated at Mediclinic City Hospital in Dubai, United Arab Emirates.

Knowing the effectiveness of IV iron infusion is essential because non-compliant pregnant women with oral iron supplementation can present with iron deficiency anemia later in their pregnancy, which can lead to unfortunate pregnancy outcomes such as those mentioned above. Therefore, an immediate IV infusion of iron is required; accordingly, understanding its effectiveness will contribute to better IV iron infusion supplementation and patient education. The hypothesis for this study is that IV iron administration would increase hemoglobin levels to a safe range (12-16 g/dL).

Aim and objectives

To investigate the effectiveness of IV iron infusion in increasing hemoglobin levels in GIDA patients, we assessed the effectiveness of IV iron infusion supplementation by the maternal age of 25-45. Moreover, the effectiveness of IV iron infusion was compared between the gestational weeks of 20-27 and 28-30 weeks. We estimated the incidence of GIDA in a tertiary-care hospital in the United Arab Emirates between 2018 and 2019.

## Materials and methods

Study design

This research was a retrospective cohort study in which the selected subjects had already been exposed to IV supplementation [18]. The data analysis was performed on anemic pregnant females aged 25-45 who were patients of Mediclinic City Hospital between 2018 and 2019. This is non-probability purposive sampling.

Setting

The data were obtained from the North Wing, Mediclinic City Hospital, where the data were sourced anonymously from hospital records of inpatient anemic pregnant females supplied with IV iron. Furthermore, all follow-up information would be readily available on the database.

Participants

All eligible participants include patients who are non-compliant to oral iron tablets or display severe anemia (hemoglobin ≤10 mmol/L) [19]. Pregnant women age group of 25-45 years old from 2018 to 2019 taking IV iron infusion during their second trimester or/and third trimester will be identified by their obstetric-gynecologist doctor and will be selected randomly. Any participants excluded include men, non-anemic pregnant women, non-pregnant women aged below 25 or above 45, or pregnant women in their first trimester.

Variables

The variables accounted for were maternal age, gestation age when IV iron infusion was administered, and the dose of IV iron. The diagnostic criteria for iron-deficiency anemia are based on a complete cell blood count with a low hemoglobin count [19]. Type of anemia as microcytic, hypochromic, and thalassemia trait ruled out.

Quantitative variables

This research was mainly based on the levels of hemoglobin reflected in the patient’s records. The document was password-protected to ensure confidentiality. IBM-SPSS for Windows version 24.0 (IBM Corp., Armonk, NY) was used for the statistical analysis of the data collected.

Statistical methods

The data were anonymously extracted from the medical records to compute the variables (hemoglobin level before IV iron infusion and after supplementation). Furthermore, the incidence was calculated using SPSS, and the mean hemoglobin level increase was compared with the recommended hemoglobin range of 12-16 g/dL [19] to determine IV iron’s effectiveness. Any missing data was identified and replaced by an estimated value from existing data.

Ethical consideration

The data extraction process ensured the anonymity of all patient information. No patient was excluded from this study based on racial, religious, or cultural backgrounds.

## Results

Participants

A total of 722 patients’ medical records were given for the study by Mediclinic City Hospital under analysis (2018-2019). However, only 40 patients met the inclusion criteria (Table 1). All patients continued until delivery.

**Table 1:** Demographic and clinical outcome of patients with iron deficiency gestational anemia admitted for intravenous iron infusion in Mediclinic City Hospital, Dubai, UAE, 2018-2019. The total number of patients’ data collected was 40. Abbreviations: Gestational week at delivery (WOD), Maternal age groups (MAG), Single administration (SA), two administrations (TA), and three or more administrations (TMA). Continuous variables are presented as mean. Categorical variables are presented as frequencies (%) At collection, the gestational week of the first admission was collected, but for analysis, they were grouped into trimesters: the second trimester (14-26 weeks) and the third trimester(26-40 weeks). The number of intravenous (IV) iron infusions reflects how many injections were given until delivery. Hemoglobin level was collected before the first admission through intravenous iron infusion, and it is expressed in grams per deciliter (g/dL).

MAG	All	WOD	Trimester	Hb level before	SA	TA	TMA	Hb level after
25-30 years	32.5%	35.4	Second	9.13(g/dL)	3	0	1	11.13(g/dL)
			Third	9.13(g/dL)	6	2	1	10.24(g/dL)
30-35 years	42.5%	37.8	Second	7.85(g/dL)	1	1	2	10.18(g/dL)
			Third	8.87(g/dL)	6	3	4	10.16(g/dL)
35-40 years	20%	37.8	Second	9.35(g/dL)	2	0	0	11.65(g/dL)
			Third	9.84(g/dL)	4	1	1	10.09(g/dL)
40-45 years	5%	26.5	Second	10.00(g/dL)	1	0	0	10.00(g/dL)
			Third	9.00(g/dL)	0	1	0	9.55(g/dL)

Descriptive data

**Figure 1:**
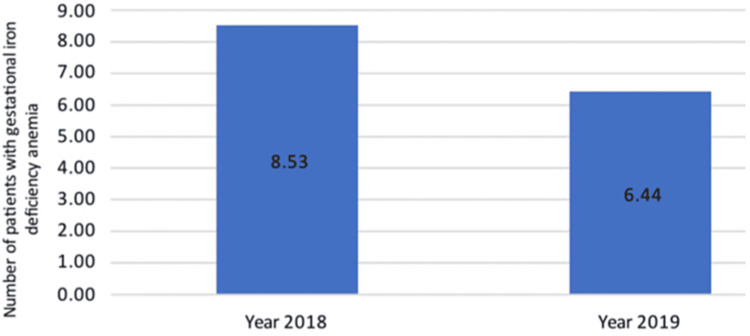
Incidence of gestational iron deficiency anemia in Mediclinic City Hospital, 2018-2019

The mean age of patients was 33.1, with the most recorded age group being 30-35 years old and the least age group being 40-45 years old (Table 1). The incidence calculated in 2018 was 8.5 per 1,000 cases of gestational iron deficiency anemia, and in 2019, it was 6.4 per 1,000 cases of gestational iron deficiency anemia (Figure 1).

**Figure 2:**
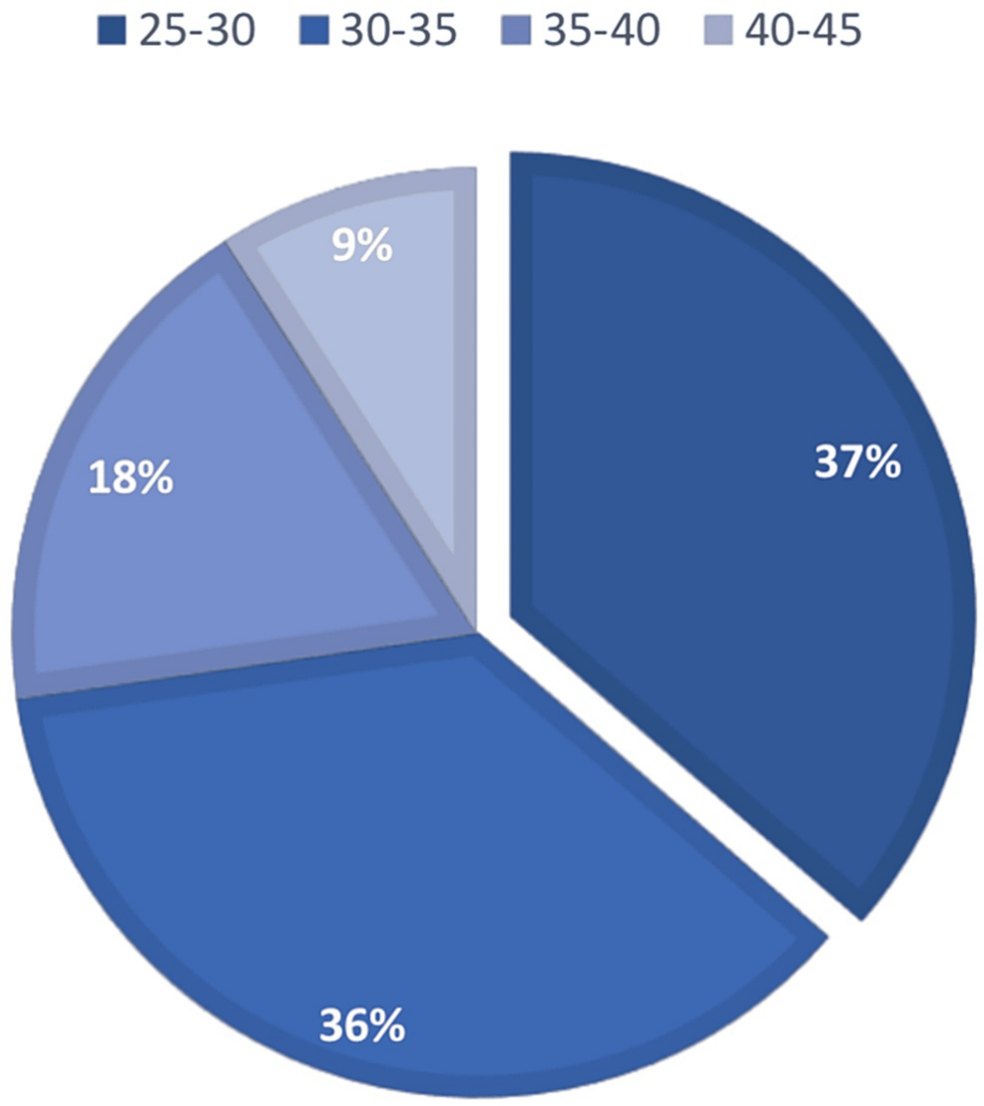
Patients admitted for gestational iron deficiency anemia in their second trimester for intravenous iron infusion at Mediclinic City Hospital Dubai, UAE, 2018- 2019

**Figure 3:**
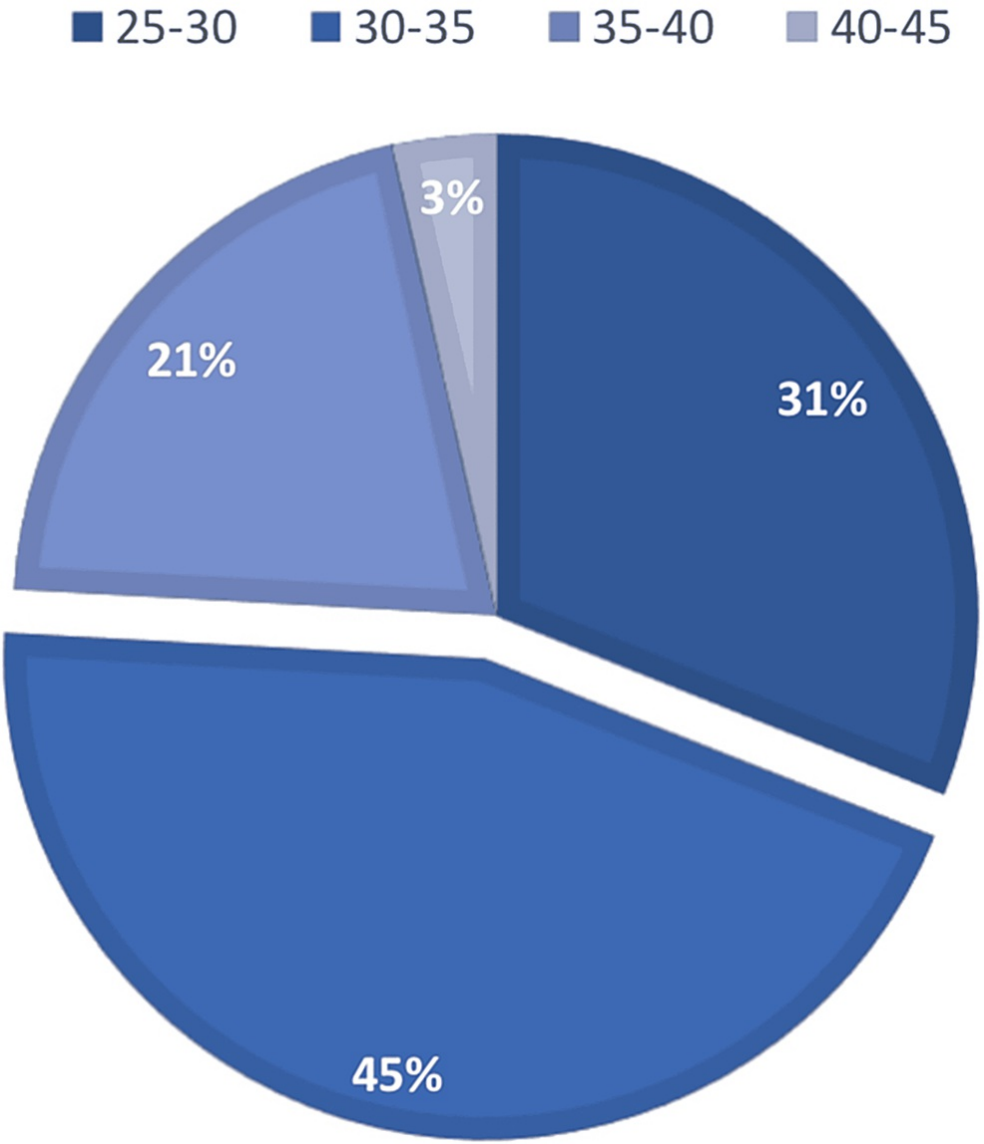
Patients admitted for gestational iron deficiency anemia in their third trimester for intravenous iron infusion at Mediclinic City Hospital Dubai, UAE, 2018-2019

Figures 2, 3 show the frequency of patients who were in their second trimester when they were admitted for their first IV iron infusion, in addition to the patients who were admitted in their third trimester. Overall, most patients admitted were in their third trimester.

The mean hemoglobin level before the intervention of IV iron infusion supplementation was 9.0 g/dL (Table 2). 

**Table 2:** Hemoglobin level mean change after administration of intravenous iron to patients with gestational iron deficiency anemia in Mediclinic City Hospital Dubai, UAE, 2018-2019. Hemoglobin recommended range is 12-16 g/dL, Standard deviation (SD) A paired sample test was applied to assess the mean difference between the hemoglobin change before and after intravenous iron infusion. A p-value of ≤0.05 was considered for statistical significance.

Hemoglobin Status	Mean	SD	P value
Hemoglobin Before	9.0	0.77	
Hemoglobin After	10.4	1.1	
Change in Hemoglobin	1.4	0.98	P<0.01

The lowest hemoglobin level recorded was 7.85 g/dL belonging to the age group 30-35 years, who were admitted in their second trimester of gestation (Table 1). The highest hemoglobin level recorded was 10 g/dL belonging to the age group 40-45 in the second trimester. There was no evident correlation between age and hemoglobin level before intervention.

The majority of patients were given only one administration of IV iron infusion supplementation, only eight patients were given two administrations, and nine patients were given three or more administrations (Table 1). Analysis revealed no relationship between the mother's age or trimester at admission, the number of administrations to the hemoglobin level, or any of these factors. The gestation week of delivery averaged 34.4 weeks. Furthermore, the age groups 25-30 years and 30-35 years had the same average of 37.8 weeks. All follow-throughs ended after the patients were delivered.

Outcome data

Hemoglobin level was recorded before delivery and after their final administration of IV iron infusion. The mean hemoglobin level was 10.4 g/dL (Table 2). The increase in hemoglobin level was significant in the age group 30-35, who were admitted in their second trimester, with a change of 2.33 g/dL.

It can be seen that the age group 30-35 who were admitted both in the second and third trimester had a mean change of 1.81 g/dL, which makes it the highest mean change between all age groups (Table 1). The average change in hemoglobin was 1.4 g/dL (Table 2).

In Table 3, the study compared the second trimester to the third trimester to see whether the change in hemoglobin would be significant to indicate that the effectiveness of IV iron infusion supplementation would differ in either trimester. The p-value recorded was 0.049, there is a statistical significance. Patients admitted in the second trimester showed a mean change of 2 g/dL and the patients admitted in the third trimester showed a mean change of 1 g/dL.

**Table 3:** Comparing the change in hemoglobin level after intravenous iron infusion supplementation between patients admitted in the second trimester and the third trimester in Mediclinic City Hospital Dubai, UAE, 2018-2019 Categorical variables are presented in frequencies. An independent samples t-test was performed to compare the groups. The p-value before rounding was 0.049141. A p-value of ≤0.05 was considered for statistical significance.

Trimester	Frequency	P value
Second trimester (%)	27.5%	
Third trimester (%)	72.5%	
Hb Change		0.049

## Discussion

To assess the effectiveness of IV iron infusion, this study looked at the change of hemoglobin after the intervention of IV iron infusion to the recommended level of 12-16 g/dL. The average hemoglobin level before intervention was 9 g/dL, and the average change after intervention was 10.4 g/dL, with a mean of 1.4 g/dL difference between before and after intervention. A paired sample test assessed the mean difference between the hemoglobin change before and after IV iron infusion. A p-value of ≤0.05 was considered for statistical significance. Reflecting the recommended level, IV iron infusion did increase hemoglobin level. However, it did not meet the recommended level range.

A study conducted in Abu Dhabi, UAE, looked into the effectiveness, tolerability, and safety of ferric carboxymaltose in the management of iron deficiency anemia in pregnant women [18]. It showed that the average hemoglobin level before the increase was 9.38 g/dL; after the intervention, it was 11.16 g/dL, which is an increase of 1.77 g/dL.

Another study assessed the efficacy of IV ferric carboxymaltose (FCM) in pregnant women and found that the median hemoglobin at the first FCM administration was 8.4 g/dL and increased to 10.7 g/dL at the time of delivery, an increase difference of 2.3 g/dL [20].

In order to assist practitioners in Dubai, UAE, this study will present updated data on the efficacy of IV iron infusion supplementation in GIDA patients. In addition, it could be used to educate pregnant women who are non-compliant with oral iron supplementation to prevent their hemoglobin levels from falling due to iron deficiency. Doctors must inform their patients, giving them knowledge about diagnosis, treatment, and heightened risk awareness [21].

This is the first study to assess the effectiveness of IV iron infusion supplementation in GIDA patients in Dubai, UAE. However, this study has limitations. The data have only been collected from a single hospital in Dubai; thus, restricting the generalizability of the findings. Furthermore, there was insufficient data in the trial to examine the time required for hemoglobin levels to return to the normal range.

This study data includes only findings from Mediclinic City Hospital in Dubai, UAE, collected over one year. Thus, the results are not generalizable to all tertiary-care hospitals in the UAE. The data available limited this study. Future studies will explore IV iron infusion supplementation, assess the effectiveness of different IV iron infusions, and determine which infusion provides maximal efficiency for each gestational age.

## Conclusions

Iron deficiency is commonly found among pregnant women. It is easily diagnosed through a blood analysis. Since pregnant women have a higher need for iron, it is imperative to advise them to take oral supplements. This study evaluated the increase in hemoglobin levels following IV iron infusion in pregnant women. It increased hemoglobin levels after the intervention. However, the increase did not meet the recommended 12-16 g/dL range.
